# Supplementary material for: Cell-type dependence of effects from transcranial electric brain stimulation
Source: PLoS Comput Biol. 2026 Oct 22;22(10):e1014895. doi: 10.1371/journal.pcbi.1014895 (PMC13722302; doi:10.1371/journal.pcbi.1014895)
Supplement: Supplementary file 1 [file pcbi.1014895.s009.pdf]

S1. Derivation of the transfer function for ball-and-sticks models

This derivation is based on the work in Pettersen et al. [2014].

Complex cable equation We start with the cable equation for a cylinder with a constant diameter d_d, which is given by

$$\lambda^2 \frac{\partial^2 V_\text{m}(x,t)}{\partial x^2} = \tau_\text{m} \frac{\partial V_\text{m}(x,t)}{\partial t} + \partial V_\text{m}(x,t) \tag{A.1}$$

where we have the neuron length constant $\lambda = \sqrt{d_\text{d} R_\text{m}/4R_\text{i}}$ and the membrane time constant $\tau_\text{m} = R_\text{m} C_\text{m}$. These constant indicate the temporal and spatial duration of an electric signal in the cable. The membrane potential V_m falls with $1/e$ after time τ_m or after the length λ [Halnes et al., 2024, p.58], depending on how much current that exits the cell through the membrane. This is decided by the electrical properties of the neuron: The specific membrane resistance (R_m), specific membrane capacitance (C_m) and inner resistivity (R_i), the specific membrane conductance, $G_\text{m} = 1/R_\text{m}$ will also be used. With dimensionless variables the cable equation can be written as

$$\frac{\partial V_\text{m}^2(X,T)}{\partial X^2} - \frac{\partial V_\text{m}(X,T)}{\partial T} - V_\text{m}(X,T) = 0 \tag{A.2}$$

where $X = x/\lambda$ is the dimensionless length and $T = t/\tau_\text{m}$ is the dimensionless time.

Our goal is to understand a neuron's response to different frequencies of an input current. Because the cable equation is linear in the subthreshold domain, each frequency-component of the input signal can be can be treated individually via Fourier. Therefore it is useful to use complex notation (bold text) where V_m is expressed in the frequency domain as

$$\mathbf{V}_\text{m} = \hat{\mathbf{V}}_\text{m}(X,W) e^{jWT} \tag{A.3}$$

where $W = \omega \tau_\text{m}$ is the dimensionless frequency ($\omega = 2\pi f$). Now $\hat{\mathbf{V}}_\text{m}$ contains both the amplitude and phase of the membrane potential, and is related to $V_\text{m}(X,T)$ via the Fourier components of the potential, given by

$$V_\text{m}(X,T) = V_{\text{m},0}(X) + \sum_{k=1}^{\infty} Re\{\hat{\mathbf{V}}_\text{m}(X,W_k) e^{jW_k T}\}. \tag{A.4}$$

If we perform the Fourier transform, using $V_\text{m} = \hat{\mathbf{V}}_\text{m} e^{jWT}$ and considering a single frequency component W_k, this can be substituted in to Eq. A.2, and we get the cable equation in complex notation, simplified to

$$\frac{d^2 \hat{\mathbf{V}}_\text{m}}{dX} - \mathbf{q}^2 \hat{\mathbf{V}}_\text{m} = 0 \tag{A.5}$$

where $\mathbf{q}^2 \equiv 1 + jW$. For a finite stick with the electrotonic length $L = l/\lambda$, where l is the stick length, the general solution Eq. A.5 can be written as

$$\hat{\mathbf{V}}_\text{m}(X,W) = \mathbf{C}_1 \cosh(\mathbf{q}L - \mathbf{q}X) + \mathbf{C}_2 \sinh(\mathbf{q}L - \mathbf{q}X). \tag{A.6}$$

Specific Solution for a dendritic stick In order to find the specific solution to the cable equation for a dendritic stick (cylinder) attached to a soma receiving an input current, we will use the boundaries with input at $X = 0$ and sealed end at $X = L$. When there is a current input in the soma, the input current creates an initial membrane potential ($\hat{\mathbf{V}}_{\text{m},0}$) in the soma which acts as the driving force for the current flow though the dendritic stick. At $X = 0$, where the dendrite is attached to the soma, the membrane potential is continuous, which gives the first boundary condition

$\hat{\mathbf{V}}_{\mathrm{m}}(X=0,W)=\hat{\mathbf{V}}_{\mathrm{m},0}$. The sealed end boundary condition means no axial current though the end of the stick. The axial current in the dendritic stick is given by

$$I_{\mathrm{i}}(x,t)=-\frac{1}{r_{\mathrm{i}}}\frac{\partial V_{\mathrm{m}}(x,t)}{\partial x} \tag{A.7}$$

where $r_{\mathrm{i}}=4R_{\mathrm{i}}/\pi d_{\mathrm{d}}^2$ is the inner resistance per unit length of cable. With complex notation and dimensionless variables this is written as

$$\hat{\mathbf{I}}_{\mathrm{i}}(X,W)=-\frac{1}{r_{\mathrm{i}}\lambda}\frac{\partial\hat{\mathbf{V}}_{\mathrm{m}}(X,T)}{\partial X}=-G_{\infty}\frac{\partial\hat{\mathbf{V}}_{\mathrm{m}}(X,T)}{\partial X} \tag{A.8}$$

where $G_{\infty}=1/r_{\mathrm{i}}\lambda$ is the infinite-stick conductance. The second boundary condition is then $\partial\hat{\mathbf{V}}_{\mathrm{m}}(X=L,W)/\partial X=0$. These two boundary conditions gives the specific solution to the cable equation for the dendritic stick as

$$\hat{\mathbf{V}}_{\mathrm{m}}(X,W)=\hat{\mathbf{V}}_{\mathrm{m},0}\frac{\cosh(\mathbf{q}L-\mathbf{q}X)}{\cosh(\mathbf{q}L)}. \tag{A.9}$$

Input admittance The initial membrane potential $\hat{\mathbf{V}}_{\mathrm{m},0}$ can be related to the input current $\hat{\mathbf{I}}_{\mathrm{in}}$ via the input admittance $\mathbf{Y}_{\mathrm{in}}=\hat{\mathbf{I}}_{\mathrm{in}}/\hat{\mathbf{V}}_{\mathrm{m},0}$. If we first consider a ball-and-stick neuron receiving an input current $\hat{\mathbf{I}}_{\mathrm{in}}$ in the soma, due to Kirchhoff's current law, $\hat{\mathbf{I}}_{\mathrm{in}}$ has to be the equal to the sum of current leaving the soma through the membrane, i.e., the somatic membrane current $\hat{\mathbf{I}}_{\mathrm{m,s}}$, and the current leaving the soma into the dendritic stick, i.e.,the axial current in the dendrite $\hat{\mathbf{I}}_{\mathrm{i}}(X,W)$ at $X=0$. The soma can be represented as an RC-circuit, which with complex notation gives the somatic membrane current density $\hat{\mathbf{i}}_{\mathrm{m,s}}$ as

$$\hat{\mathbf{i}}_{\mathrm{m,s}}=G_{\mathrm{m}}\mathbf{q}^2\hat{\mathbf{V}}_{\mathrm{m},0}. \tag{A.10}$$

A spherical soma with diameter d_{s} gives the area of the soma $A_s=\pi d_{\mathrm{s}^2}$, and the input admittance $\mathbf{Y}_{\mathrm{s}}=\hat{\mathbf{I}}_{\mathrm{m,s}}/\hat{\mathbf{V}}_{\mathrm{m},0}$ will therefore be given as

$$\mathbf{Y}_{\mathrm{s}}=\pi d_{\mathrm{s}}^2G_{\mathrm{m}}\mathbf{q}^2. \tag{A.11}$$

The current leaving the soma going into the dendrite is given by axial current $\hat{\mathbf{I}}_{\mathrm{i}}(X,W)$ at $X=0$. By inserting the specific solution to $\hat{\mathbf{V}}_{\mathrm{m}}(X,W)$ from Eq. A.9 into the equation for the axial current in Eq. A.8 we get

$$\hat{\mathbf{I}}_{i}(X,W)=\hat{\mathbf{V}}_{\mathrm{m},0}\mathbf{q}G_{\infty}\frac{\sinh(\mathbf{q}L-\mathbf{q}X)}{\cosh(\mathbf{q}L)}, \tag{A.12}$$

which gives the input admittance of the dendritic stick $\mathbf{Y}_{\mathrm{in,d}}=\hat{\mathbf{I}}_{i}(X=0,W)/\hat{\mathbf{V}}_{\mathrm{m},0}$ as

$$\mathbf{Y}_{\mathrm{in,d}}=\mathbf{q}G_{\infty}\tanh(\mathbf{q}L). \tag{A.13}$$

For an infinite stick $\lim_{L\to\infty}\tanh(\mathbf{q}L)\to 1$, the infinite-stick admittance $\mathbf{Y}_{\infty}(W)=\mathbf{q}G_{\infty}$.

By Kirchoff's current law, the total input admittance for the ball-and-stick is given by $\mathbf{Y}_{\mathrm{in}}=\mathbf{Y}_{\mathrm{s}}+\mathbf{Y}_{\mathrm{d}}$. If the neuron instead consist of N number of dendritic sticks, the input current will spread to all N sticks, and the input admittance is therefore now affected by the sum of all individual dendritic stick admittances. This gives

$$\mathbf{Y}_{\mathrm{in}}=\mathbf{Y}_{\mathrm{s}}+\sum_{n=1}^{N}\mathbf{Y}_{\mathrm{d},n}=\mathbf{Y}_{\mathrm{s}}+\sum_{n=1}^{N}\mathbf{Y}_{\infty,n}\tanh(\mathbf{q}L_n). \tag{A.14}$$

Dipole moment from a dendritic stick In order to find an expression for the dipole moment from somatic current input we must look at the membrane currents through the dendritic stick. This is given as

$$i_{\mathrm{m,d}} = -\frac{\partial I_{\mathrm{i}}(x,t)}{\partial x} = \frac{1}{r_{\mathrm{i}}}\frac{\partial^2 V_{\mathrm{m}}(x,t)}{\partial x^2}, \quad \text{(A.15)}$$

which with complex notation is expressed as

$$\hat{\mathbf{i}}_{\mathrm{m,d}}(X,W) = -\frac{1}{\lambda}\frac{\partial \hat{\mathbf{I}}(X,W)}{\partial X} = \frac{1}{r_{\mathrm{i}}\lambda^2}\frac{\partial^2 \hat{\mathbf{V}}_{\mathrm{m}}(X,W)}{\partial X^2} = g_{\mathrm{m}}\frac{\partial^2 \hat{\mathbf{V}}_{\mathrm{m}}(X,W)}{\partial X^2}. \quad \text{(A.16)}$$

With the specific solution for $\hat{\mathbf{V}}_{\mathrm{m}}(X,W)$ in Eq. A.9, we get the membrane current in the dendritic stick as

$$\hat{\mathbf{i}}_{\mathrm{m,d}}(X_d,W) = g_{\mathrm{m}}\mathbf{q}^2\frac{\cosh(\mathbf{q}L_d - \mathbf{q}X_d)}{\cosh(\mathbf{q}L_d)}\hat{\mathbf{V}}_{\mathrm{m},0}. \quad \text{(A.17)}$$

The dipole moment is the sum of all membrane currents multiplied with their position [Halnes et al., 2024]. With a continuous cable, the complex dipole moment from the dendritic stick with a sealed end is given by the integral

$$\hat{\mathbf{p}}_{\mathrm{d}}(W) = \lambda^2\int_0^{L_{\mathrm{d}}}\hat{\mathbf{i}}_{\mathrm{m,d}}(X,W)X\,\mathrm{d}X = \lambda G_\infty\hat{\mathbf{V}}_{\mathrm{m},0}\left[1 - \frac{1}{\cosh(\mathbf{q}L_d)}\right], \quad \text{(A.18)}$$

and have the direction of the dendritic stick.

Transfer function Our goal is to find a transfer function relating the dipole moment in the z-direction ($\hat{\mathbf{p}}_z$) and the input current $\hat{\mathbf{I}}_{\mathrm{in}}$ in the soma as

$$\mathbf{T}_p = \frac{\hat{\mathbf{p}}_z}{\hat{\mathbf{I}}_{\mathrm{in}}} \quad \text{(A.19)}$$

The dipole moment from a dendritic stick $\hat{\mathbf{p}}_d$, given in Eq A.18 has the direction of the stick. This dipole moment projection onto the z-axis is further given by $\hat{\mathbf{p}}_d\cos(\theta)$ where θ is the angle between the direction of the stick and the z-direction. For N number of dendritic stick, the total dipole moment in the z-direction $\hat{\mathbf{p}}_z$ is then

$$\hat{\mathbf{p}}_z = \sum_{n=1}^{N}\hat{\mathbf{p}}_{d,n}\cos(\theta_n) = \hat{\mathbf{V}}_{\mathrm{m},0}\sum_{n=1}^{N}\lambda_n G_{\infty,n}\left[1 - \frac{1}{\cosh(\mathbf{q}_n L_n)}\right]\cos(\theta_n). \quad \text{(A.20)}$$

This gives the transfer function

$$\mathbf{T}_p = \frac{1}{\mathbf{Y}_{\mathrm{in}}}\sum_{n=1}^{N}\left(\lambda_n G_{\infty,n}\left[1 - \frac{1}{\cosh(\mathbf{q}_n L_n)}\right]\cos(\theta_n)\right), \quad \text{(A.21)}$$

since $\mathbf{Y}_{\mathrm{in}} = \hat{\mathbf{I}}_{\mathrm{in}}/\hat{\mathbf{V}}_{\mathrm{in}}$.

Transfer function for Ball-and-Two-Sticks: We consider the case where the neuron has two dendritic sticks point in opposite directions along the z-axis and have identical neural parameters and identical dendrite diameter d_{d}, we then have

- **Directions:** $\theta_1 = 0^\circ \implies \cos\theta_1 = 1$, and $\theta_2 = 180^\circ \implies \cos\theta_2 = -1$.
- **Parameters:** $\lambda_1 = \lambda_2$, $G_{\infty,1} = G_{\infty,2}$, $Y_{\infty,1} = Y_{\infty,2}$, $\mathbf{q}_1 = \mathbf{q}_2$.

The transfer function can then be simplified to

$$\mathbf{T}_p = \frac{\lambda G_\infty}{\mathbf{Y}_{\text{in}}} \left(\frac{1}{\cosh(\mathbf{q}L_2)} - \frac{1}{\cosh(\mathbf{q}L_1)} \right), \tag{A.22}$$

where the total input admittance is:

$$\mathbf{Y}_{\text{in}} = \mathbf{Y}_{\text{s}} + \mathbf{Y}_\infty(\tanh(\mathbf{q}L_1) + \tanh(\mathbf{q}L_2)).$$

This full expression for the transfer function Ball-and-Two-Sticks neuron is then

$$\mathbf{T}_p = \frac{\lambda G_\infty \left(\frac{1}{\cosh(\mathbf{q}L_2)} - \frac{1}{\cosh(\mathbf{q}L_1)} \right)}{\mathbf{Y}_{\text{s}} + \mathbf{Y}_\infty(\tanh(\mathbf{q}L_1) + \tanh(\mathbf{q}L_2))}. \tag{A.23}$$

To gain insight into how different neural geometries affect the transfer function, $\mathbf{T}_p^{\text{s}}$ can be written out as

$$\mathbf{T}_p = \frac{\frac{d_{\text{d}}^2}{4R_{\text{i}}} \left(\frac{1}{\cosh(\mathbf{q}L_1)} - \frac{1}{\cosh(\mathbf{q}L_2)} \right)}{\left(\frac{d_{\text{s}}^2}{R_{\text{m}}} \mathbf{q}^2 \right) + \left(\mathbf{q} \sqrt{\frac{d_{\text{d}}^3}{4R_{\text{i}}R_{\text{m}}}} \right) (\tanh(\mathbf{q}L_1) + \tanh(\mathbf{q}L_2))}. \tag{A.24}$$

Following the same approach, this can be generalized to an arbitrary number of dendrites with individual lengths, diameters, and angles θ_i. This gives,

$$\mathbf{T}_p = \frac{\sum_{i=1}^{N} \lambda_i G_{\infty,i} \left(1 - \frac{1}{\cosh(\mathbf{q}L_i)} \right) \cos(\theta_i)}{\mathbf{Y}_{\text{s}} + \sum_{i=1}^{N} \mathbf{Y}_{\text{d},i}}, \tag{A.25}$$

where the different components are listed in Tab A1.

References

Halnes, G., Ness, T.V., Næss, S., Hagen, E., Pettersen, K.H., Einevoll, G.T., 2024. Electric Brain Signals: Foundations and Applications of Biophysical Modeling. 1 ed., Cambridge University Press, Cambridge, UK. doi:10.1017/9781009039826.

Pettersen, K.H., Lindén, H., Tetzlaff, T., Einevoll, G.T., 2014. Power Laws from Linear Neuronal Cable Theory: Power Spectral Densities of the Soma Potential, Soma Membrane Current and Single-Neuron Contribution to the EEG. PLOS Computational Biology 10, e1003928. URL: https://journals.plos.org/ploscompbiol/article?id=10.1371/journal.pcbi.1003928, doi:10.1371/journal.pcbi.1003928. publisher: Public Library of Science.

Table A1: List of symbols and parameters in alphabetical order.

Symbol	Default value (Unit)	Description
$C_{\rm m}$	0.01 pF/µm^2	specific membrane capacitance
$d_{\rm d}$	2 µm	dendrite diameter
$d_{\rm s}$	20 µm	soma diameter
f	$1 - 2000$ Hz	frequency
$G_{\rm m} = 1/R_{\rm m}$	0.333 pS/µm^2	specific membrane conductance
$G_\infty = \pi d_{\rm d}^2/4R_{\rm i}\lambda$	2.09 nS	infinite-stick conductance
$L = l/\lambda$	(1)	electrotonic length
l	(µm)	stick length
$\mathbf{q} = \sqrt{1 + jW}$	(1)	frequency dependence
$R_{\rm i}$	1.5 MΩ µm	inner resistivity
$R_{\rm m}$	3 MΩµm^2	specific membrane resistance
$T = t/\tau_{\rm m}$	(1)	dimensionless time
$W = \omega\tau_{\rm m}$	(1)	dimensionless frequency
$X = x/\lambda$	(1)	dimensionless position
$\mathbf{Y}_{\rm in}$	(S)	input admittance
$\mathbf{Y}_{\rm s} = \pi d_{\rm s}^2 G_{\rm m}\mathbf{q^2}$	(S)	soma admittance
$\mathbf{Y}_{\rm d} = \mathbf{q}G_\infty \tanh(\mathbf{q}L)$	(S)	dendrite admittance
$\mathbf{Y}_\infty = \mathbf{q}G_\infty$	(S)	infinite–stick admittance
$\lambda = \sqrt{d_{\rm d}R_{\rm m}/4R_{\rm i}}$	1 mm	neuron length constant (DC)
$\tau_{\rm m} = R_{\rm m}C_{\rm m}$	30 ms	membrane time constant
$\omega = 2\pi f$	(rad/s)	angular frequency

Selection of relevant symbols and parameters for the transfer function explaining dipole moment per input current. Table adapted from Pettersen et al. [2014].
