# Supplementary material for: Cell-type dependence of effects from transcranial electric brain stimulation
Source: PLoS Comput Biol. 2026 Oct 22;22(10):e1014895. doi: 10.1371/journal.pcbi.1014895 (PMC13722302; doi:10.1371/journal.pcbi.1014895)
Supplement: Supplementary file 2 [file pcbi.1014895.s011.pdf]

Dear Editor;

We hereby resubmit our manuscript “Cell-type dependence of effects from transcranial electric brain stimulation” by Susanne Dahle, Gaute T. Einevoll, and Torbjørn V. Ness for publication in PLoS Computational Biology.

We would like to thank the editor and the three referees for their thoughtful comments and efforts in reviewing the manuscript. In response to their criticisms, we have substantially revised and improved the manuscript. Among the major changes are a new result section (Section 2.5) and a new figure (Figure 12) demonstrating how the reciprocity-based approach can potentially be useful also in non-linear cases. Furthermore, we have substantially expanded the Discussion regarding modelling assumptions, and how our results might be applied in future studies of the effect of transcranial electric stimulation at the network level.

Our responses to the reviewers are marked blue.

Changes in the manuscript are marked red.

With these changes we hope that the revised manuscript is found suitable for publication in PLoS Computational Biology.

Sincerely,

Torbjørn V. Ness

on behalf of the authors

Editor:

The reviewers appreciated the overall analysis, including the analytical formula for morphology-dependence of frequency response (eq. 9). Nevertheless, the interpretation at the network level is complicated by the non-linear behaviour of neurons in the living brain (because of synaptic conductance fluctuations, which in particular change the dendritic model [mentioned in the manuscript], as well as subthreshold voltage-dependent channels), in addition to the relatively small impact of the tES on the membrane potential of individual cells. These qualifications need to be addressed in your revision, as well as the other remarks made by the reviewers.

We are glad the reviewers appreciated the overall analysis. We fully agree that our results by themselves are not sufficient to predict network-level effects of tES, and we have substantially extended our analysis and discussion regarding this important point. Below are our point-wise response to each of the points raised by the reviewers.

Reviewer #1:

The authors provide a useful characterization of tES effects on neurons of different types, demonstrate the use of inverse theorem to readily simulate/calculate the effects, and derive insights about how tES response depends on morphological features that differ between neurons. The study is well written and has important applications in simulating tES relevant to clinical treatment, but would benefit from a few additional checks and refinements.

We thank the reviewer for these kind words.

Major Comments:

1. Given how interneurons vary in morphology and thus relevant tES effects as we saw in figure 1 and 7, then instead of lumping interneurons, figure 8 would be more informative if structured like figure 7 but showing results across all cells of a particular type for each of the 5 types. Similarly, figure 1C,D and figure 2 should parse out the results to subtypes of interneurons instead of lumping them together.

We agree that this is in principle a good suggestion, and we recognize that it is not optimal to lump very different interneurons together. Note, however, that although we have chosen five example cell models, this does not reflect the number of cell classes that are present in the database that we used, which has 55 different morphological cell types. Among interneurons, the inhibitory cell types in the database are Martinotti Cells (MC), Bitufted Cells (BTC), Double Bouquet Cells (DBC), Bipolar Cells (BP), Neurogliaform Cells (NGC), Large Basket Cells (LBC), Nest Basket Cells (NBC), Small Basket Cells (SBC), and Chandelier Cells (ChC). These cell types also vary depending on the layer from layer II/III, IV, V, and VI. In addition, layer I has its own inhibitory cell types, and among the excitatory cells, each layer has its own group of cell types. For a summary of the different cell types, see Figure 2 in Markram et al., 2015.

We did not find a good and clean way of visualizing the responses of all 55 different morphological cell types within one figure. We have investigated different approaches for merging inhibitory cell types into larger subgroups to reduce the number of classes, based on molecular markers (PV+, SST+, VIP+, etc), given for the cell types in Table 1 by Markram et al., 2015, however, this resulted in very fuzzy and not mutually exclusive groups. We also did not succeed in creating consistent and meaningful groups based on morphological features. We would therefore prefer to keep the grouping as it is.

2. The study would benefit from validating/comparing the results on a few published models of human pyramidal neurons and possibly also interneurons, which have some key morphological differences compared to rodents. This will demonstrate the generalization of the results and increase the relevance to human brain research and applications.

We agree that using human cell models would be a nice demonstration that our results generalize and are useful. We have therefore added a new figure which rely on a human layer 3 pyramidal cell model. The same new figure is also used to address the next comment, see below.

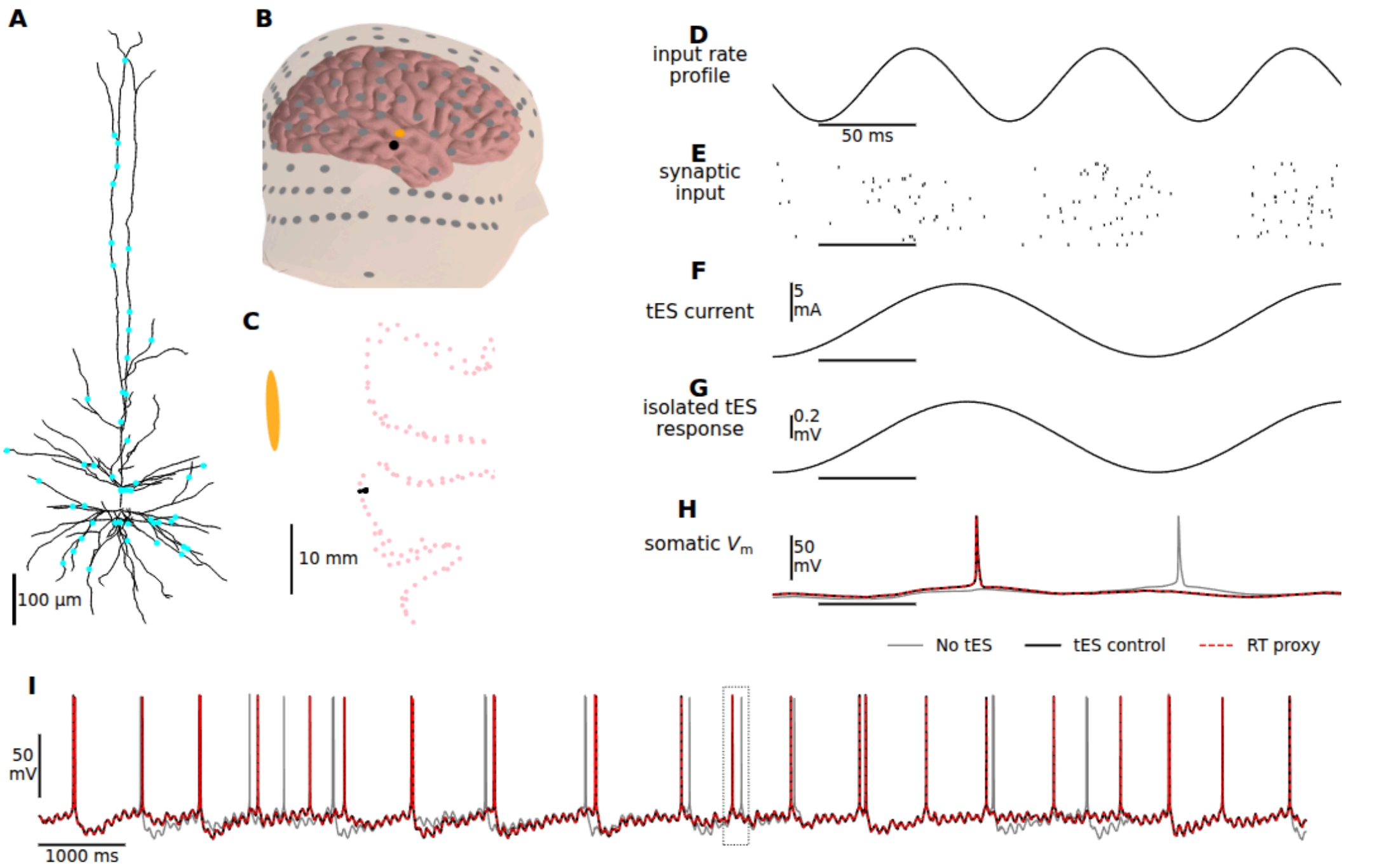

Fig 12. RT-based proxy can give accurate predictions for non-linear neural activity. A: A human layer 3 pyramidal cell model from the Allen Brain Atlas with active perisomatic conductances. The cell model has 50 conductance-based synapses uniformly distributed over the morphology (cyan dots). **B:** The New York Head model [48], with the chosen neuron location in the middle temporal gyrus marked by the black dot, and the closest tES/EEG electrode marked by the orange ellipse. **C:** Points on the local cortical surface plane close to the neuron are shown as pink dots, and the closest tES/EEG electrode as an orange ellipse. The (very small) neuron morphology is shown in black. **D:** The synapses are activated with a sinusoidal rate profile of 10 Hz. **E:** The synaptic input to the model. **F:** A current with a frequency of 5 Hz and an amplitude of 5 mA is injected through the tES/EEG electrode (orange ellipse in panel C). **G:** By inserting the (downscaled) tES current (panel F) into the soma of a passive version of the cell, and calculating the resulting (rescaled) EEG signal, we obtain the isolated tES response of the passive cell. The effect of this tES can be introduced into the active cell model receiving conductance-based synaptic input as an additional somatic current input. **H:** Somatic membrane potential response of the cell model for three different cases: (i) synaptic input and no tES (gray line), (ii) the exact same synaptic input with tES (black line), and (iii) the exact same synaptic input, but the effect of tES approximated through the reciprocity-based approach as an additional somatic current input (red dashed line). Note that the red and black lines are almost perfectly overlapping, obscuring the black line. **I:** The same as panel H, but for a broader time window. The dotted square correspond to the time window in panels D-H.

3. Authors assume the neurons are in subthreshold potential and passive regime for the reciprocity theorem to apply, but what about the case of tES applied to the living brain, where the potential varies and is more depolarized? Authors should at least discuss this matter and predict more explicitly how their results apply to the more depolarized case.

This is a very important point, and we agree that it warrants a more detailed discussion. We have added a new Results section (Section 2.5) dedicated to this point, with a corresponding new figure (Figure 12). The point of the figure is to illustrate how one can apply the reciprocity-based approach in a more realistic setting. An active (spiking) human pyramidal cell model is receiving conductance-based synaptic input, while also under the influence of tES. We demonstrate that the reciprocity-based approach to predicting the effect of tES is accurate, also in this case. We argue in the discussion that this indicates that the RT-based approach can be used to accurately incorporate the effect of tES in, for example, point-neuron network simulations.

Minor Comments:

1. Fig 3 – to form a better picture, authors could show results for intermediate frequencies such as 50 and/or 100 Hz

We have now added a new panel for 100 Hz to Figure 3 as a new panel B.

2. Authors assumed passive model, but should discuss the relevance of subthreshold effect of ion channels (e.g. on resonance or voltage), and any dendritic channel differences in the different neuron types

We have now improved the discussion regarding this. Around line 592, we write:

"The reciprocity-based approach relies on linearity, and we therefore mostly used passive cell models. Such cell models are unable to fire action potentials, which is acceptable for our purposes since our study primarily considered subthreshold tES-responses. We confirmed that the active and passive versions of the cell models in general gave very similar results to subthreshold tES, although some cell models exhibited differences below about 10 Hz (Fig S1). The Ih channel has previously been demonstrated to play a key role in low-frequency subthreshold modulation of membrane potentials, and can give rise to pronounced peaks in power spectra both in extracellular potentials generated by cells [42, 43], and in neural responses to tES [44]. A detailed investigation of the effect of the Ih conductance was outside the scope of the present study, but note that linearized, so-called quasi-active, versions of the Ih conductance are highly accurate [42– 44, 59]. Should it be deemed necessary, the subthreshold effect of Ih on tES responses can therefore be studied using the same reciprocity-based approach as was used here. "

3. Beyond morphological properties, authors can check if passive electrical properties are useful features to predict tES response

This is an interesting suggestion. For a passive cell model, any combination of passive conductances can in general be lumped together into a single equivalent passive conductance. This means that the relevant passive electrical parameters are the (potentially location dependent) membrane resistance (R_m), the membrane capacitance (C_m) and the axial resistance (R_i). For a clean analysis, we investigated this using the analytical formula and found that the effect of a uniform change in the membrane resistance (R_m) was negligible at all frequencies. At high frequencies, the axial resistance and membrane capacitance had a certain effect, but still weaker than the effect of the asymmetry factor (AF) and dendritic diameter (d_d).

We have added this information to Fig S8:

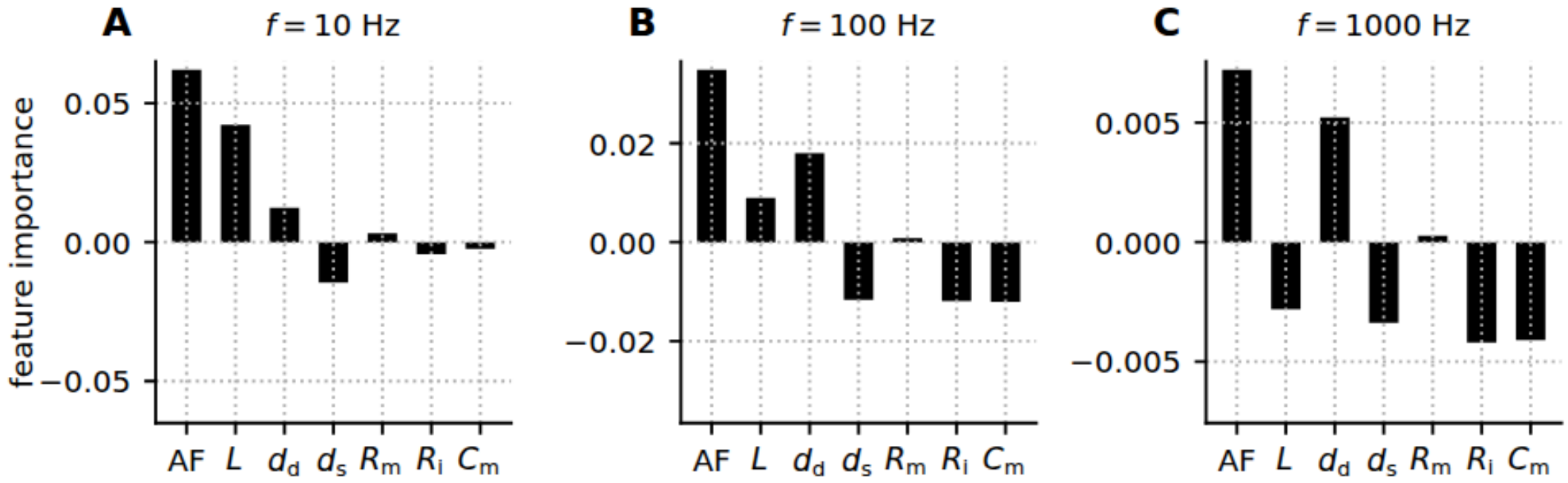

Fig S8: Feature importance values from linear regression on idealized ball-and-two-sticks models. The linear regression model included seven selected properties to predict soma V_m amplitudes of ball-and-two-sticks models, resulting from applied electric field oriented in the z-direction perpendicular to the cortical surface. **A:** At $f = 10$ Hz, the regression model achieved R^2=0.87. The asymmetry factor and total length had the highest absolute feature importance values. **B:** At $f = 100$ Hz, the regression model achieved R^2=0.77. The asymmetry factor and dendritic diameter had the highest absolute feature importance values. **C:** At $f = 1000$ Hz, the regression model achieved R^2=0.68. The asymmetry factor still had the highest absolute feature importance value, while total length had the opposite effect as for 10 Hz and 100 Hz. The parameters were independently and randomly chosen with uniform probability density within certain ranges. For the morphological parameters, the total length was in the range 200-1000 μm, the asymmetry factor was between 0 and 1, the somatic diameter was in the range 5-20 μm, and the dendritic diameter was in the range 0.5-2 μm. For the passive electrophysiological parameters, the membrane resistance was in the range 15000-60000 Ωcm^2, the axial resistance was in the range 50-200 Ωcm, and the membrane capacitance was in the range 0.5-2 μF/cm^2. In total, 5000 cell models were included.

4. Figure 2 and corresponding results would benefit from a statistical test to support statements

We agree that statistical tests would strengthen the analysis. We have therefore modified Figure 2 and the corresponding text to include the appropriate statistical tests, which supports the claims made in the text.

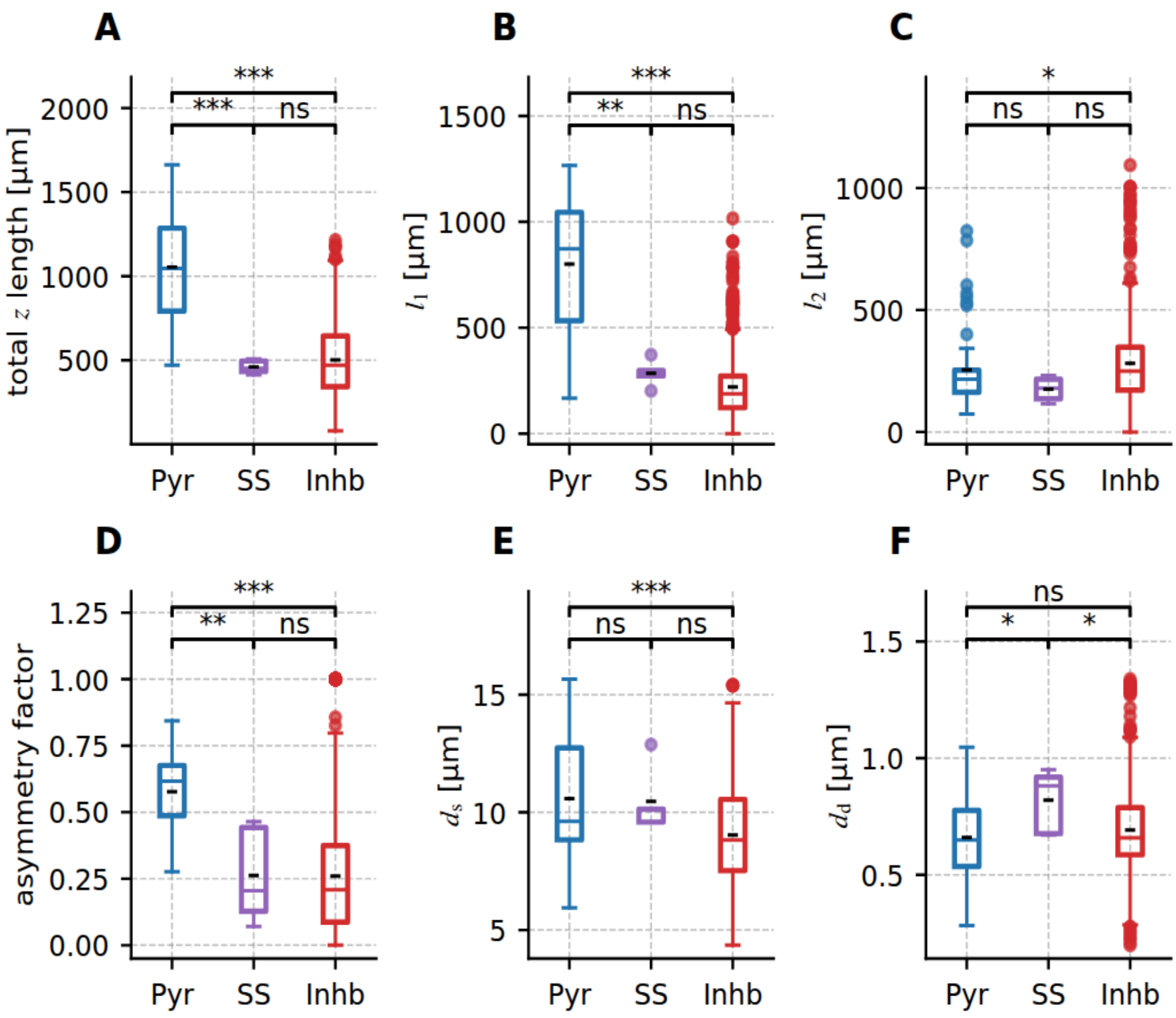

Fig 2. Morphological properties are highly cell-type specific. Box plots of different morphological properties for three classes of neurons, including pyramidal cells (Pyr), stellate cells (SS), and inhibitory interneurons (Inhb). **A:** The total length in the z-direction, calculated as the distance between the upper and lower endpoints. **B:** The length of the neuron above the soma (l_1), i.e, the distance from the soma to the upper endpoint. **C:** The length of the neuron below the soma (l_2), i.e, the distance from the soma to the lower endpoint. **D:** Asymmetry factor, defined as $|l_1 - l_2|/(l_1 + l_2)$. **E:** Diameter of the soma compartment. **F:** Average diameter of the compartments pointing in z-direction. See Methods for more details about how the properties were found. Median marked with a colored line spanning the entire box, mean marked with a shorter black line. The boxes represent the interquartile (IQR) range (25th–75th percentile), containing the middle 50% of the data. Whiskers extend to 1.5 × IQR, and dots denote outliers beyond 1.5 × IQR. Statistically significant differences between groups where calculated using two-sided Mann-Whitney U tests, where *** indicate $p<0.001$, ** indicate $p<0.01$, * indicate $p<0.05$, and ns indicate no significant difference.

5. The captions of Fig2E/F are mixed

We thank the reviewer for pointing out this mistake. It has now been corrected.

6. Fig 11 – caption is missing part B

We thank the reviewer for pointing out this mistake. It has now been corrected.

Reviewer #2:

In this manuscript authors investigate how electrical stimulation may affect the membrane potential of neurons with different shapes. The main result is that "the susceptibility of a neuron to tES is directly proportional to its dipole moment for somatic current input". Then they build on this and (some previous results of the Einevoll group) to show how the dipole moment depends on the neuron morphology. The analytical expression of the neuron transfer function (eq9) is a nice contribution. This leads to some interesting conclusions about the asymmetry of neurons

that shapes the dipole moment and how that makes neurons sensitive to electrical stimulation. For example longer neurons are more sensitive to stimulation frequency. These are all interesting observations. I have three main objections that reduce the strength of this contribution.

We are glad to hear that the reviewer appreciate our manuscript.

1/ Reciprocity theorem works for linear systems and neurons have all kinds of non-linearities. So it is not clear to what extent these observations will hold in a realistic setting.

This is indeed the most obvious limitation of the reciprocity-based approach and as such it deserves thorough consideration. To better address this issue, we have added a new section in Results (Section 2.5), and a new Figure 12:

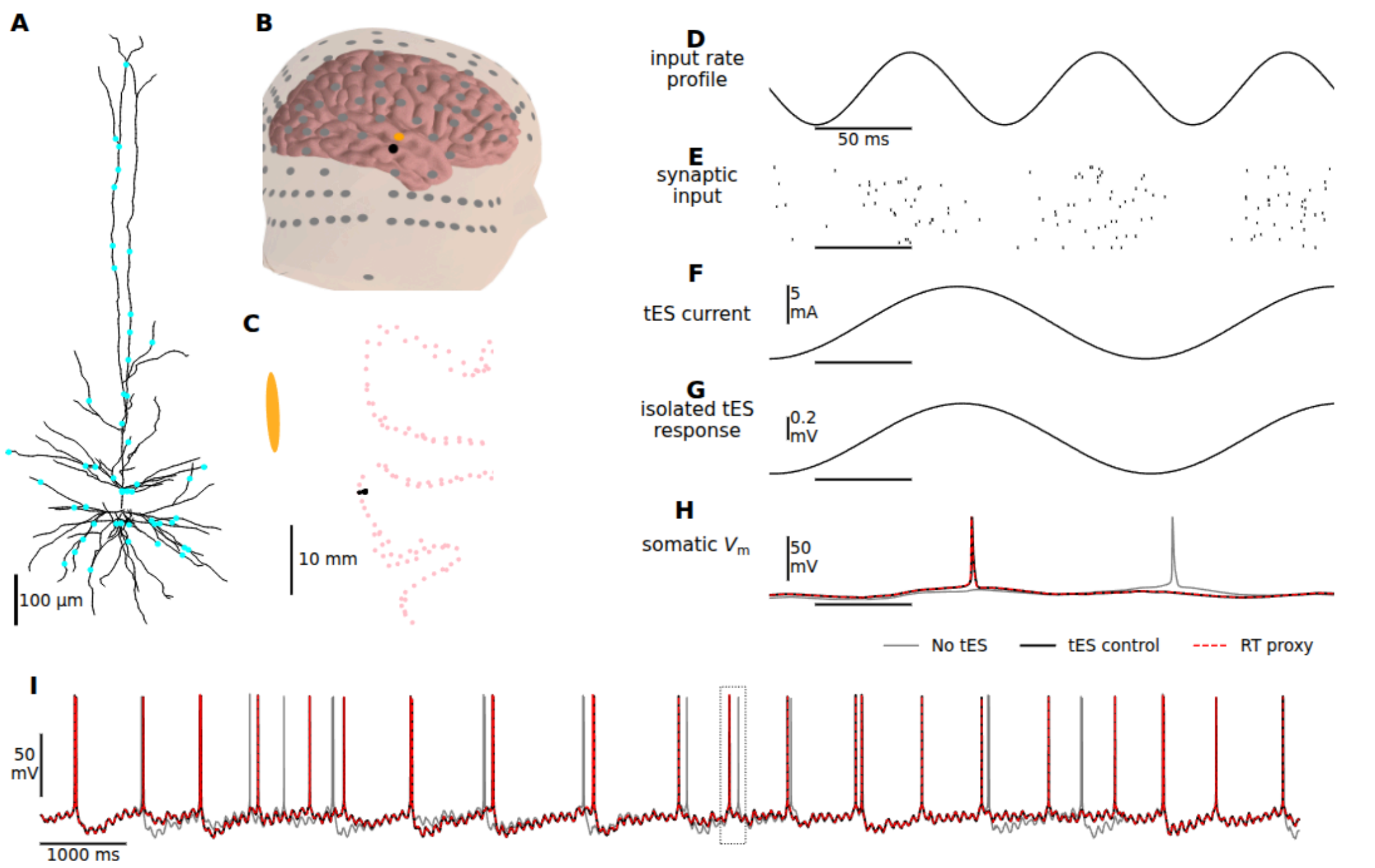

Fig 12. RT-based proxy can give accurate predictions for non-linear neural activity. A: A human layer 3 pyramidal cell model from the Allen Brain Atlas with active perisomatic conductances. The cell model has 50 conductance-based synapses uniformly distributed over the morphology (cyan dots). **B:** The New York Head model [48], with the chosen neuron location in the middle temporal gyrus marked by the black dot, and the closest tES/EEG electrode marked by the orange ellipse. **C:** Points on the local cortical surface plane close to the neuron are shown as pink dots, and the closest tES/EEG electrode as an orange ellipse. The (very small) neuron morphology is shown in black. **D:** The synapses are activated with a sinusoidal rate profile of 10 Hz. **E:** The synaptic input to the model. **F:** A current with a frequency of 5 Hz and an amplitude of 5 mA is injected through the tES/EEG electrode (orange ellipse in panel C). **G:** By inserting the (downscaled) tES current (panel F) into the soma of a passive version of the cell, and calculating the resulting (rescaled) EEG signal, we obtain the isolated tES response of the passive cell. The effect of this tES can be introduced into the active cell model receiving conductance-based synaptic input as an additional somatic current input. **H:** Somatic membrane potential response of the cell model for three different cases: (i) synaptic input and no tES (gray line), (ii) the exact same synaptic input with tES (black line), and (iii) the exact same synaptic input, but the effect of tES approximated through the reciprocity-based approach as an additional somatic current input (red dashed line). Note that the red and black lines are almost perfectly overlapping, obscuring the black line. **I:** The same as panel H, but for a broader time window. The dotted square correspond to the time window in panels D-H.

2/ More critically, I do not agree with the claim of the authors that "These findings help us better understand the effect of tES at the fundamental level of single-cell membrane potential responses, and provide us with helpful rules-of-thumb". The reasons are :
(a) The contribution of the activity of a neuron type depends on network state and neuron's connectivity
(b) Networks react differently to different frequencies, therefore different frequencies may result in very different states and thereby complicate the contribution of individual neuron types.

We understand the concern of the reviewer, and agree that network state and connectivity are very important for understanding tES responses. However, as the direct effect of stimulation is on individual neurons, the understanding of network responses must build on an accurate understanding of single-cell responses. The present manuscript attempts to characterize such individual single-cell responses so that these can be used later to study network effects. We have changed the quoted sentence to better reflect this, around line 497:

"The direct effect of tES is on neurons individually, and our findings help to better understand the effect the stimulation has on the membrane potentials of individual neurons. In a simulation of network responses to tES, these individual effects can be added directly to all neuron models included in the simulation. "

Furthermore, the new Figure 12 and corresponding text also adds to the discussion of this point.

3/ The effect on the membrane potential even in the best case is very small. Typically neurons show fluctuations of the magnitude of ~5mV. In that background noise increasing the mean of a neuron by 0.2mV (in the best case) is not likely to do much to the network. The best we can take from the paper is that external stimulation may induce different amounts of E/I currents - overall effect then is subject to the network connectivity and state.

The isolated effect of tES on single-cell membrane potentials is indeed expected to be very small: We mostly considered an electric field of 1 mV/mm, which–as the reviewer points out–resulted in amplitude responses up to on the order of 0.2 mV. It might seem surprising that such small fluctuations should affect neural activity, however, neural effects of field strengths as low as 0.2 mV/mm are well documented, see for example:

- Liu A, Vöröslakos M, Kronberg G, Henin S, Krause MR, Huang Y, et al. Immediate neurophysiological effects of transcranial electrical stimulation. Nat Commun. 2018;9(1):5092. https://doi.org/10.1038/s41467-018-07233-7
- Tran H, Shirinpour S, Opitz A. Effects of transcranial alternating current stimulation on spiking activity in computational models of single neocortical neurons. Neuroimage. 2022;250:118953. https://doi.org/10.1016/j.neuroimage.2022.118953
- Reato D, Rahman A, Bikson M, Parra LC. Low-intensity electrical stimulation affects network dynamics by modulating population rate and spike timing. J Neurosci. 2010;30(45):15067–79. https://doi.org/10.1523/JNEUROSCI.2059-10.2010
- Johnson L, Alekseichuk I, Krieg J, Doyle A, Yu Y, Vitek J, et al. Dose-dependent effects of transcranial alternating current stimulation on spike timing in awake nonhuman primates. Sci Adv. 2020;6(36):eaaz2747. https://doi.org/10.1126/sciadv.aaz2747
- Alekseichuk I, Wischnewski M, Opitz A. A minimum effective dose for (transcranial) alternating current stimulation. Brain Stimul. 2022;15(5):1221–2. https://doi.org/10.1016/j.brs.2022.08.018
- Zhao Z, Shirinpour S, Tran H, Wischnewski M, Opitz A. intensity- and frequency-specific effects of transcranial alternating current stimulation are explained by network dynamics. J Neural Eng. 2024;21(2):10.1088/1741-2552/ad37d9. https://doi.org/10.1088/1741-2552/ad37d9

The neural effect from tES is often explained in terms of stochastic resonance and the sizable ambient noise level in vivo: If a large number of neurons are slightly more likely to fire an action potential during one phase of a sinusoidal stimuli, and slightly less likely to fire an action potential during the other phase, then even a weak sinusoidal stimuli can introduce an

oscillation in the network activity. We have added a new paragraph regarding this around line 635:

"The isolated effect of tES on single-cell membrane potentials is small: We mostly considered an electric field of 1 mV/mm, which resulted in amplitude responses on the order of up to 0.2 mV. This is substantially lower than background membrane potential fluctuations which are expected under *in vivo* conditions, and it might therefore seem counterintuitive that such small tES responses should affect neural activity. Note, however, that neural effects from field strengths as low as 0.2 mV/mm are well documented [15, 19, 33, 58, 59] with so-called stochastic resonance effects being a suggested explanation [10].

That tES can affect spiking activity is also illustrated in the new Figure 12 where a neuron is receiving background synaptic input which occasionally brings it close enough to spiking that a difference of ~0.3 mV is sufficient to either initiate or prevent spikes.

Some specific comments:

- Figure 1: I understand that these results were obtained when the neurons were silent. But in a realistic situation neurons would receive local inputs when the tES arrives. How would the results change if neurons were to have an ongoing activity and local inputs along their dendrites. Note that in ongoing activity dendritic electronic length would change. Moreover, different regions of the dendrite could be more or less active depending on the brain state (especially for the pyramidal neurons where we see the maximal response). Then the same concern extends to the analytical expressions.

Figure 12 illustrates that the reciprocity-approach can also be applied when non-linear effects are present. Regardless of whether the neuron model is active or not, the effect of tES can, in principle, be added to the membrane potential. However, the magnitude of this effect depends on the linear response of the neuron membrane around the working point, and this will in general depend on the state of the neuron. For the example in Figure 12, this variation in the linear response where small and as a consequence the RT-method gave almost perfect result. However, in general, as the reviewer points out, ongoing activity will affect the effective dendritic electronic length, and this happens because conductance-based synaptic input modulates the effective membrane conductance.

For the purpose of calculating LFP signals, such synapse-induced effective changes in membrane conductances have recently been takes into account to enable the use of linear methods, see Hagen et al. (2022) and Meneghetti et al. (2025). In these studies, arbitrary levels of conductance-based synaptic background input are used to estimate the effective membrane conductance, also under different states (Meneghetti et al., 2025). The same approach could be used for reciprocity-based calculation of tES responses, also for the analytic formulas. We have added around line 606:

"Another non-linearity stems from conductance-based synaptic input (as opposed to the linear current-based synaptic input). Importantly, such synaptic input can change the effective membrane conductance of cells [52], which can again affect their tES-sensitivity. For a given level of conductance-based synaptic input, it is, however, possible to calculate the resulting effective membrane conductance, and this approach was recently successfully applied to enable linear calculation of electric brain signals from complex networks of non-linear cell models [59, 60]. Notably, Meneghetti et al. (2025) used the biophysically detailed large-scale model of mouse visual cortex developed by the Allen Institute [61], and achieved high accuracy with a linear model that separated the neural activity into ”high-input states” and ”low-input states” [60]. The same approach could be applied to reciprocity-based calculation of tES

responses by modifying the leak conductance to reflect the effective membrane conductance, either in biophysically detailed cell models or the analytic formula (eq 9). This would reveal how single-cell tES responses depend on the background-level of synaptic input."

- I think the figure 3 is redundant. By now it is clear that individual features would not predict any property of a neuron and we should consider multiple properties together.

In response to a suggestion from Reviewer 1 (minor point 1), we have modified this figure, and we therefore do not want to remove it at this point.

- Line 242: Should it not be axial resistance of the membrane?

Line 242 in the original submission reads: "Here, tau_m = R_m C_m is the membrane time constant, and ω = 2πf is the angular frequency." Here tau_m is (as stated) the membrane time constant, R_m is the membrane resistance, and C_m the membrane capacitance. The axial resistance does not enter in this expression. We notice, however, that R_m and C_m were not properly defined, and we have now done so, and added a citation to the book on biophysics by Koch (1999) covering the underlying theory. Line 250 now reads:

"Here, tau_m = *R*_m *C*_m is the membrane time constant, *R*_m is the membrane resistance, *C*_m is the membrane capacitance, and *ω* = 2*πf* is the angular frequency [52]."

Minor

- Lines 161-165: 'is' is missing at a crucial point in this sentence

We thank the reviewer for pointing this out, and we have rewritten the sentence to make it more readable.

Reviewer #3:

Thanks for the opportunity to review this paper. The manuscript describes a previously presented framework for assessing the impact of transcranial electrical stimulation on neuron models. The paper extends the previous work by applying the approach to a number of different types of neurons, revealing insights into the frequency dependence of tES in these neurons, which I believe would be of interest to the readers of PLOS Computational Biology.

We thank the reviewer for the positive evaluation of our manuscript.

Below I have some minor comments to help clarify the manuscript:

1. The reader may benefit from more explanation of the quasi-static assumption.

To clarify for the reader that we are relying on the quasi-electrostatic approximation, we have added around Line 689:

"This modelling framework relies on the quasi-electrostatic approximation of Maxwell's equations [21], meaning that terms with time derivatives of the magnetic field are neglected. This approximation appears to be well-justified for the relatively low frequencies considered here below a few thousand hertz [21, 72, 73]."

2. Could the authors explain/reference why the frequency range chosen was 1Hz to 2kHz - and if the latter relevant in applications of tES?

The broad frequency range was chosen so that our results could be relevant to tES of arbitrary frequency. Traditional tES tend to use quite low frequencies, below 100 Hz or so, but the new and merging temporal interference stimulation uses frequencies of 2 kHz and above. Around line 81, we have added:

"We considered a broad frequency range, from 1 Hz to 2 kHz (see Methods), so that our results have relevance for different types of tES, ranging from transcranial direct current stimulation (tDCS) [35], spanning the frequency range of transcranial alternating current stimulation (tACS, <100 Hz) [10], and towards temporal interference stimulation at high frequencies (1 kHz and upwards) [40]."

3. The authors explain that they assumed one particular orientation of neurons relative to the field. Although this is discussed and other orientations were simulated, in the real brain would this have a big impact on the influence of tES on neural activity?

The orientation of the electric field relative to the neurons is indeed expected to have a big impact on how tES affects neural activity, both for the amplitude and the phase of the response. We now more explicitly discuss this around line 546:

"Note that we here focused on amplitude responses, but the phase of the responses can also be important: For pyramidal neurons with the same orientation relative to a tES electrode, we expect the cells to be depolarized/hyperpolarized in phase, and the tES will therefore be synchronizing. However, populations of pyramidal neurons with opposite orientation relative to the tES electrode, will experience the opposite effect and their responses will be out of phase. For interneurons with less pronounced asymmetry, even neighboring interneurons might experience the opposite effect from the same tES, depending on their exact morphology [37]. The effect of the orientation of the local electric field relative to the neural orientations will, therefore, be important to take into account in future network studies of tES."

4. Although the rational for removing the active currents is given and the authors show that passive and active are broadly similar, there are some clear differences shown in Figure S1. Could the authors give more motivation for this simplification?

We have expanded the discussion regarding this point, and around line 592 we now write:

"The reciprocity-based approach relies on linearity, and we therefore used passive cell models. We confirmed that the active and passive versions of the cell models in general gave very similar results to subthreshold tES, although some cell models exhibited differences below about 10 Hz (Fig S1). The Ih channel has previously been demonstrated to play a key role in low-frequency subthreshold modulation of membrane potentials, and can give rise to pronounced peaks in power spectra both in extracellular potentials generated by cells [42, 43], and in neural responses to tES [44]. A detailed investigation of the effect of the Ih conductance was outside the scope of the present study, but note that linearized, so-called quasi-active, versions of the Ih conductance are highly accurate [42– 44, 59]. Should it be deemed necessary, the subthreshold effect of Ih on tES can therefore be studied using the same reciprocity-based approach as was used here."

5. I would like to see more discussion on the significance of these results with respect to specific paradigms/experiments utilising tES?

Our goal with this manuscript was not to mimic any specific paradigm or experiment, but rather to characterize the frequency-dependent tES-sensitivity of different cell-types across a broad frequency range, such that the results are relevant for different tES paradigms. We have now made this more explicit in the text, around line 81:

"We considered a broad frequency range, from 1 Hz to 2 kHz (see Methods), so that our results have relevance for different types of tES, ranging from transcranial direct current stimulation (tDCS) [35], spanning the typical frequency range of transcranial alternating current stimulation (tACS, <100 Hz) [10], and towards temporal interference stimulation at high frequencies (1 kHz and upwards) [40]."

Also in the Discussion, around line 500, we have added:

"Instead of focusing on a specific tES paradigm, we used a broad frequency-range so that our results are relevant for different types of tES, like tDCS, tACS, and temporal interference stimulation."

6. Figure 1: the order of the legend entries could be consistent for ease of comparison. Also, the caption reads “Example cells from panel B are marked.” But it is not made clear how they are marked. Do the authors refer to the bold lines, if so these are not easy to identify.

The order has now been fixed so that it is consistent between panel C and D. We have also rewritten the caption to clarify that the data from the example cells in panel B is also for comparison shown in panel C as bold lines. These lines have also been made more visible in panel C.

7. Figures: Could the authors consider consistency in the colour coding throughout the paper, particularly in reserving the three colours for pyramidal, inhibitory and stellate cells.

We have gone over all figures and made the color scheme more consistent, and in particular reserved three colors for pyramidal, inhibitory, and stellate cells.

8. Figure 6: the caption only refers to part A-C of the figure, and D-F are not described. Although this is presumably for conciseness, I think the reader would benefit from a brief explanation that D-F mirror A-C but for a short neuron.

We thank the reviewer for pointing out this mistake which has now been fixed.
