# Supplementary material for: Cell-type dependence of effects from transcranial electric brain stimulation
Source: PLoS Comput Biol. 2026 Oct 22;22(10):e1014895. doi: 10.1371/journal.pcbi.1014895 (PMC13722302; doi:10.1371/journal.pcbi.1014895)
Supplement: Peer review history

# Peer review: Decision Letter 0

**DOI:** 10.1371/journal.pcbi.1014895.r001

**Authors:** Lyle Graham

23 Jun 2026

Cell-type dependence of effects from transcranial electric brain stimulation

PLOS Computational Biology

Dear Dr. Ness,

Please submit your revised manuscript by Aug 23 2026 11:59PM. If you will need more time than this to complete your revisions, please reply to this message or contact the journal office at ploscompbiol@plos.org. When you're ready to submit your revision, log on to https://www.editorialmanager.com/pcompbiol/ and select the 'Submissions Needing Revision' folder to locate your manuscript file.

Please include the following items when submitting your revised manuscript:

* A letter that responds to each point raised by the editor and reviewer(s). You should upload this letter as a separate file labeled 'Response to Reviewers'. This file does not need to include responses to formatting updates and technical items listed in the 'Journal Requirements' section below.

* A marked-up copy of your manuscript that highlights changes made to the original version. You should upload this as a separate file labeled 'Revised Manuscript with Track Changes'.

* An unmarked version of your revised paper without tracked changes. You should upload this as a separate file labeled 'Manuscript'.

Kind regards,

Lyle J. Graham

Section Editor

PLOS Computational Biology

**Journal Requirements:**

If the reviewer comments include a recommendation to cite specific previously published works, please review and evaluate these publications to determine whether they are relevant and should be cited. There is no requirement to cite these works unless the editor has indicated otherwise.

1) We ask that a manuscript source file is provided at Revision. Please upload your manuscript file as a .doc, .docx, .rtf or .tex. If you are providing a .tex file, please upload it under the item type u2018LaTeX Source Fileu2019 and leave your .pdf version as the item type u2018Manuscriptu2019.

2) Please upload all main figures as separate Figure files in .tif or .eps format. For more information about how to convert and format your figure files please see our guidelines:

https://journals.plos.org/ploscompbiol/s/figures

3) Please upload a copy of Figure Fig 1-11 which you refer to in your text on pages 4, 5, 7, 9, 10, 11, 14, and 15. Or, if the figure is no longer to be included as part of the submission please remove all reference to it within the text.

4) We notice that your supplementary Figures, and Tables are included in the manuscript file. Please remove them and upload them with the file type 'Supporting Information'. Please ensure that each Supporting Information file has a legend listed in the manuscript after the references list.

5) We have amended your Competing Interest statement to comply with journal style. We kindly ask that you double check the statement and let us know if anything is incorrect.

**Reviewers' comments:**

Reviewer's Responses to Questions

**Comments to the Authors:**

**Please note here if the review is uploaded as an attachment.**

Reviewer #1: The authors provide a useful characterization of tES effects on neurons of different types, demonstrate the use of inverse theorem to readily simulate/calculate the effects, and derive insights about how tES response depends on morphological features that differ between neurons. The study is well written and has important applications in simulating tES relevant to clinical treatment, but would benefit from a few additional checks and refinements.

Major Comments:

1. Given how interneurons vary in morphology and thus relevant tES effects as we saw in figure 1 and 7, then instead of lumping interneurons, figure 8 would be more informative if structured like figure 7 but showing results across all cells of a particular type for each of the 5 types.

Similarly, figure 1C,D and figure 2 should parse out the results to subtypes of interneurons instead of lumping them together.

2. The study would benefit from validating/comparing the results on a few published models of human pyramidal neurons and possibly also interneurons, which have some key morphological differences compared to rodents. This will demonstrate the generalization of the results and increase the relevance to human brain research and applications.

3. Authors assume the neurons are in subthreshold potential and passive regime for the reciprocity theorem to apply, but what about the case of tES applied to the living brain, where the potential varies and is more depolarized? Authors should at least discuss this matter and predict more explicitly how their results apply to the more depolarized case.

Minor Comments:

1. Fig 3 – to form a better picture, authors could show results for intermediate frequencies such as 50 and/or 100 Hz

2. Authors assumed passive model, but should discuss the relevance of subthreshold effect of ion channels (e.g. on resonance or voltage), and any dendritic channel differences in the different neuron types

3. Beyond morphological properties, authors can check if passive electrical properties are useful features to predict tES response

4. Figure 2 and corresponding results would benefit from a statistical test to support statements

5. The captions of Fig2E/F are mixed

6. Fig 11 – caption is missing part B

Reviewer #2: In this manuscript authors investigate how electrical stimulation may affect the membrane potential of neurons with different shapes. The main result is that "the susceptibility of a neuron to tES is directly proportional to its dipole moment for somatic current input". Then they build on this and (some previous results of the Einevoll group) to show how the dipole moment depends on the neuron morphology. The analytical expression of the neuron transfer function (eq9) is a nice contribution. This leads to some interesting conclusions about the asymmetry of neurons that shapes the dipole moment and how that makes neurons sensitive to electrical stimulation. For example longer neurons are more sensitive to stimulation frequency. These are all interesting observations. I have three main objections that reduce the strength of this contribution.

1/ Reciprocity theorem works for linear systems and neurons have all kinds of non-linearities. So it is not clear to what extent these observations will hold in a realistic setting.

2/ More critically, I do not agree with the claim of the authors that "These findings help us better understand the effect of tES at the fundamental level of single-cell membrane potential responses, and provide us with helpful rules-of-thumb". The reasons are :

(a) The contribution of the activity of a neuron type depends on network state and neuron's connectivity

(b) Networks react differently to different frequencies, therefore different frequencies may result in very different states and thereby complicate the contribution of individual neuron types.

3/ The effect on the membrane potential even in the best case is very small. Typically neurons show fluctuations of the magnitude of ~5mV. In that background noise increasing the mean of a neuron by 0.2mV (in the best case) is not likely to do much to the network.

The best we can take from the paper is that external stimulation may induce different amounts of E/I currents - overall effect then is subject to the network connectivity and state.

Some specific comments:

- Figure 1: I understand that these results were obtained when the neurons were silent. But in a realistic situation neurons would receive local inputs when the tES arrives. How would the results change if neurons were to have an ongoing activity and local inputs along their dendrites. Note that in ongoing activity dendritic electronic length would change. Moreover, different regions of the dendrite could be more or less active depending on the brain state (especially for the pyramidal neurons where we see the maximal response). Then the same concern extends to the analytical expressions.

- I think the figure 3 is redundant. By now it is clear that individual features would not predict any property of a neuron and we should consider multiple properties together.

- Line 242: Should it not be axial resistance of the membrane?

Minor

- Lines 161-165: 'is' is missing at a crucial point in this sentence

Reviewer #3: Thanks for the opportunity to review this paper. The manuscript describes a previously presented framework for assessing the impact of transcranial electrical stimulation on neuron models. The paper extends the previous work by applying the approach to a number of different types of neurons, revealing insights into the frequency dependence of tES in these neurons, which I believe would be of interest to the readers of PLOS Computational Biology.

Below I have some minor comments to help clarify the manuscript:

1. The reader may benefit from more explanation of the quasi-static assumption.

2. Could the authors explain/reference why the frequency range chosen was 1Hz to 2kHz - and if the latter relevant in applications of tES?

3. The authors explain that they assumed one particular orientation of neurons relative to the field. Although this is discussed and other orientations were simulated, in the real brain would this have a big impact on the influence of tES on neural activity?

4. Although the rational for removing the active currents is given and the authors show that passive and active are broadly similar, there are some clear differences shown in Figure S1. Could the authors give more motivation for this simplification?

5. I would like to see more discussion on the significance of these results with respect to specific paradigms/experiments utilising tES?

6. Figure 1: the order of the legend entries could be consistent for ease of comparison. Also, the caption reads “Example cells from panel B are marked.” But it is not made clear how they are marked. Do the authors refer to the bold lines, if so these are not easy to identify.

7. Figures: Could the authors consider consistency in the colour coding throughout the paper, particularly in reserving the three colours for pyramidal, inhibitory and stellate cells.

8. Figure 6: the caption only refers to part A-C of the figure, and D-F are not described. Although this is presumably for conciseness, I think the reader would benefit from a brief explanation that D-F mirror A-C but for a short neuron.

**********

**Have the authors made all data and (if applicable) computational code underlying the findings in their manuscript fully available?**

The PLOS Data policy requires authors to make all data and code underlying the findings described in their manuscript fully available without restriction, with rare exception (please refer to the Data Availability Statement in the manuscript PDF file). The data and code should be provided as part of the manuscript or its supporting information, or deposited to a public repository. For example, in addition to summary statistics, the data points behind means, medians and variance measures should be available. If there are restrictions on publicly sharing data or code —e.g. participant privacy or use of data from a third party—those must be specified.

Reviewer #1: Yes

Reviewer #2: Yes

Reviewer #3: Yes

**********

PLOS authors have the option to publish the peer review history of their article (what does this mean?). If published, this will include your full peer review and any attached files.

If you choose “no”, your identity will remain anonymous but your review may still be made public.

**Do you want your identity to be public for this peer review?**  For information about this choice, including consent withdrawal, please see our Privacy Policy.

Reviewer #1: No

Reviewer #2: No

Reviewer #3: No

[NOTE: If reviewer comments were submitted as an attachment file, they will be attached to this email and accessible via the submission site. Please log into your account, locate the manuscript record, and check for the action link "View Attachments". If this link does not appear, there are no attachment files.]

**Figure resubmission:**

**Reproducibility:**

?>

# Author comment: Author response to Decision Letter 1

**DOI:** 10.1371/journal.pcbi.1014895.r002

25 Aug 2026

Attachment Submitted filename: response_letter.pdf

# Peer review: Decision Letter 1

**DOI:** 10.1371/journal.pcbi.1014895.r003

**Authors:** Lyle Graham

12 Oct 2026

Dear Dr. Ness,

We are pleased to inform you that your manuscript 'Cell-type dependence of effects from transcranial electric brain stimulation' has been provisionally accepted for publication in PLOS Computational Biology.

Before your manuscript can be formally accepted you will need to complete some formatting changes, which you will receive in a follow up email. A member of our team will be in touch with a set of requests.

Please note that your manuscript will not be scheduled for publication until you have made the required changes, so a swift response is appreciated.

IMPORTANT: The editorial review process is now complete. PLOS will only permit corrections to spelling, formatting or significant scientific errors from this point onwards. Requests for major changes, or any which affect the scientific understanding of your work, will cause delays to the publication date of your manuscript.

Should you, your institution's press office or the journal office choose to press release your paper, you will automatically be opted out of early publication. We ask that you notify us now if you or your institution is planning to press release the article. All press must be co-ordinated with PLOS.

Thank you again for supporting Open Access publishing; we are looking forward to publishing your work in PLOS Computational Biology.

Best regards,

Lyle J. Graham

Section Editor

PLOS Computational Biology

***********************************************************

Reviewer's Responses to Questions

**Comments to the Authors:**

**Please note here if the review is uploaded as an attachment.**

Reviewer #1: The authors have addressed all my comments.

Regarding major point 1 - I'm fine with their explanation, and suggest authors add a sentence to the results/discussion to support the choice of lumping interneurons - by conveying that results for the different types did not add to what they show when lumping all interneurons.

Reviewer #2: I am happy with the revision. Authors have done a very good job in addressing my concerns.

Reviewer #3: The authors have addressed all of my comments.

**********

**Have the authors made all data and (if applicable) computational code underlying the findings in their manuscript fully available?**

The PLOS Data policy requires authors to make all data and code underlying the findings described in their manuscript fully available without restriction, with rare exception (please refer to the Data Availability Statement in the manuscript PDF file). The data and code should be provided as part of the manuscript or its supporting information, or deposited to a public repository. For example, in addition to summary statistics, the data points behind means, medians and variance measures should be available. If there are restrictions on publicly sharing data or code —e.g. participant privacy or use of data from a third party—those must be specified.

Reviewer #1: None

Reviewer #2: Yes

Reviewer #3: Yes

**********

PLOS authors have the option to publish the peer review history of their article (what does this mean?). If published, this will include your full peer review and any attached files.

If you choose “no”, your identity will remain anonymous but your review may still be made public.

**Do you want your identity to be public for this peer review?**  For information about this choice, including consent withdrawal, please see our Privacy Policy.

Reviewer #1: No

Reviewer #2: No

Reviewer #3: No

# Editor report: Acceptance letter

**DOI:** 10.1371/journal.pcbi.1014895.r004

**Authors:** Lyle Graham

PCOMPBIOL-D-26-00930R1

Cell-type dependence of effects from transcranial electric brain stimulation

Dear Dr Ness,

I am pleased to inform you that your manuscript has been formally accepted for publication in PLOS Computational Biology. Your manuscript is now with our production department and you will be notified of the publication date in due course.

The corresponding author will soon be receiving a typeset proof for review, to ensure errors have not been introduced during production. Please review the PDF proof of your manuscript carefully, as this is the last chance to correct any errors. Please note that major changes, or those which affect the scientific understanding of the work, will likely cause delays to the publication date of your manuscript.

Soon after your final files are uploaded, unless you have opted out, the early version of your manuscript will be published online. The date of the early version will be your article's publication date. The final article will be published to the same URL, and all versions of the paper will be accessible to readers.

For Research, Software, and Methods articles, you will receive an invoice from PLOS for your publication fee after your manuscript has reached the completed accept phase. If you receive an email requesting payment before acceptance or for any other service, this may be a phishing scheme. Learn how to identify phishing emails and protect your accounts at https://explore.plos.org/phishing.

Thank you again for supporting PLOS Computational Biology and open-access publishing. We are looking forward to publishing your work!

With kind regards,

Sharmila Kamatchi

PLOS Computational Biology | Carlyle House, Carlyle Road, Cambridge CB4 3DN | United Kingdom ploscompbiol@plos.org | Phone +44 (0) 1223-442824 | ploscompbiol.org | @PLOSCompBiol
